# Trifunctional Kinetic Regulation Enables Low‐Defect Solution Grown Single Crystals for X‐Ray Detection

**DOI:** 10.1002/advs.75517

**Published:** 2026-05-30

**Authors:** Hui Zhang, Zihan Wang, Zhenyu Wang, Changmao Wan, Zheng Liang, Huifen Xu, Yuanbo Ma, Yupeng Liu, Xu Pan, Jiajiu Ye

**Affiliations:** ^1^ Institute of Solid‐State Physics Hefei Institutes of Physical Science Chinese Academy of Science Hefei China; ^2^ University of Science and Technology of China Hefei China

**Keywords:** Cs_4_PbI_6_, kinetic regulation, single crystals, trifunctional, X‐ray detection

## Abstract

All‐inorganic 0D material exhibits excellent stability and shows great potential in the field of X‐ray detection. However, their practical performance is limited by intrinsic point defects and solvent incorporation. Herein, we propose a universal trifunctional kinetic regulation strategy utilizing iodoacetic acid (IAA) as a multifunctional additive to dynamically regulate crystal growth. Through a synergistic mechanism, the carboxyl group (‐COOH) coordinates with undercoordinated Pb^2+^ to suppress deep‐level traps, released iodide ions (I^−^) compensate for potential iodine vacancies (V_I_), while the bulky ‐CH_2_I group provides steric hindrance against solvent incorporation. This molecular engineering approach yields Cs_4_PbI_6_ single crystals that exhibit a resistivity of 1.95 × 10^10^ Ω cm, and a remarkably enhanced carrier mobility‐lifetime product (µτ) of 2.18 × 10^−4^ cm^2^ V^−1^. X‐ray detectors based on these crystals achieve a high sensitivity of 897.4 µC Gy_air_ cm^−2^ and a low detection limit of 29.81 nGy_air_ s^−1^ under an electric field of 1000 V cm^−1^ for 80 keV X‐ray detection, alongside robust operational and environmental stability. This work provides a molecular‐level design strategy for high‐performance perovskite single‐crystal X‐ray detectors.

## Graphical abstract

This work presents a molecular‐level kinetic regulation strategy employing iodoacetic acid (IAA) to simultaneously suppress point defects and solvent inclusion in solution‐grown Cs_4_PbI_6_ single crystals. The obtained crystals demonstrate significantly enhanced charge transport properties, enabling X‐ray detectors with high sensitivity, low noise, and remarkable operational stability.

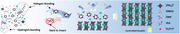

## 1 Introduction

Metal halide perovskites have rapidly advanced to the forefront of radiation‐detection research owing to their excellent performance and low manufacturing cost [1, 2, 3, 4, 5, 6, 7, 8]. Among these, all‐inorganic perovskites, such as CsPbBr_3_ [9, 10, 11, 12], Cs_2_AgBiBr_6_ [13, 14, 15], and Cs_3_Bi_2_I_9_ [16, 17, 18], have attracted significant attention due to their superior environmental stability compared to organic–inorganic hybrid counterparts. These materials benefit from strong ionic bonds and high atomic numbers (Z), ensuring efficient X‐ray attenuation. However, high dark current and operational instability, often exacerbated by ion migration under applied electric fields, remain critical challenges for many perovskite‐based detectors, particularly those with 3D architectures.

To address these limitations, 0D perovskites have emerged as a compelling alternative. Their distinct crystal framework, comprising isolated metal‐halide octahedra separated by alkali‐metal cations, significantly suppresses ion migration by introducing high activation energy barriers [19, 20, 21, 22, 23, 24]. Furthermore, the wide bandgap (3.0–4.0 eV) intrinsically limits thermally generated carriers, enabling ultralow dark currents. Previous reports have demonstrated that high‐quality Cs_4_PbI_6_ bulk single crystals grown from solution exhibit exceptional long‐term stability and high sensitivity in rigid X‐ray detectors [22]. Nevertheless, fully realizing the intrinsic advantages of Cs_4_PbI_6_ requires precise control over its crystallization pathway. Impurity phases such as CsI and the narrow‐bandgap CsPbI_3_ can readily form during synthesis, acting as detrimental defects that significantly elevate dark current and induce baseline drift [25]. While processing strategies, such as temperature‐controlled annealing, have proven effective in suppressing these impurities in polycrystalline films, they remain insufficient for producing the high‐purity, low‐defect single crystals required for optimal performance.

Specifically, in 0D perovskites, the [PbI_6_]^4−^ octahedra act as isolated islands. When an iodide ion vacates its lattice site to form a vacancy, the ensuing lattice relaxation and reconstruction are relatively minor. Consequently, the energy barrier that must be overcome to form an V_I_ is very low. Furthermore, the growth of 0D Cs_4_PbI_6_ involves the stacking of these isolated [PbI_6_]^4^^−^ octahedra. Solvent molecules (e.g., DMSO, DMF) form stable coordination complexes with Pb^2+^ and serve as fundamental units for crystal nucleation in the early stages [26]. If crystallization kinetics are not strictly regulated, the rapidly closing lattice exerts a caging effect, trapping these coordinated solvent molecules directly within the voids between the forming octahedra and preventing their effective expulsion [27, 28, 29]. Previous strategies, including the use of monofunctional additives like formic (FAH) or acetic acid (HAc), have moderately improved the crystallization process [30], but fail to simultaneously address these interconnected issues.

Herein, we introduce a kinetic regulation strategy utilizing iodoacetic acid (IAA) as a dynamic, multifunctional additive to overcome these intertwined challenges. The unique molecular structure of IAA enables a trifunctional mechanism that actively governs the crystallization process. Specifically, the ‐COO^−^ strongly coordinates with Pb^2+^ to passivate deep‐level traps and suppressing ion migration, while the I^−^ directly compensates for volatile iodine vacancies (V_I_), thereby mitigating shallow traps. Concurrently, the ‐CH_2_I moiety provides steric hindrance, effectively excluding solvent molecules from the crystal lattice and minimizing solvent‐related defects. The resulting Cs_4_PbI_6_ single crystals exhibit significantly reduced defect densities and enhanced charge transport properties. X‐ray detectors fabricated from these crystals demonstrate excellent sensitivity of 897.4 µC Gy_air_ cm^−2^, highlighting their potential as promising candidates for high‐performance optoelectronic detectors.

## 2 Results and Discussion

**FIGURE 1:**
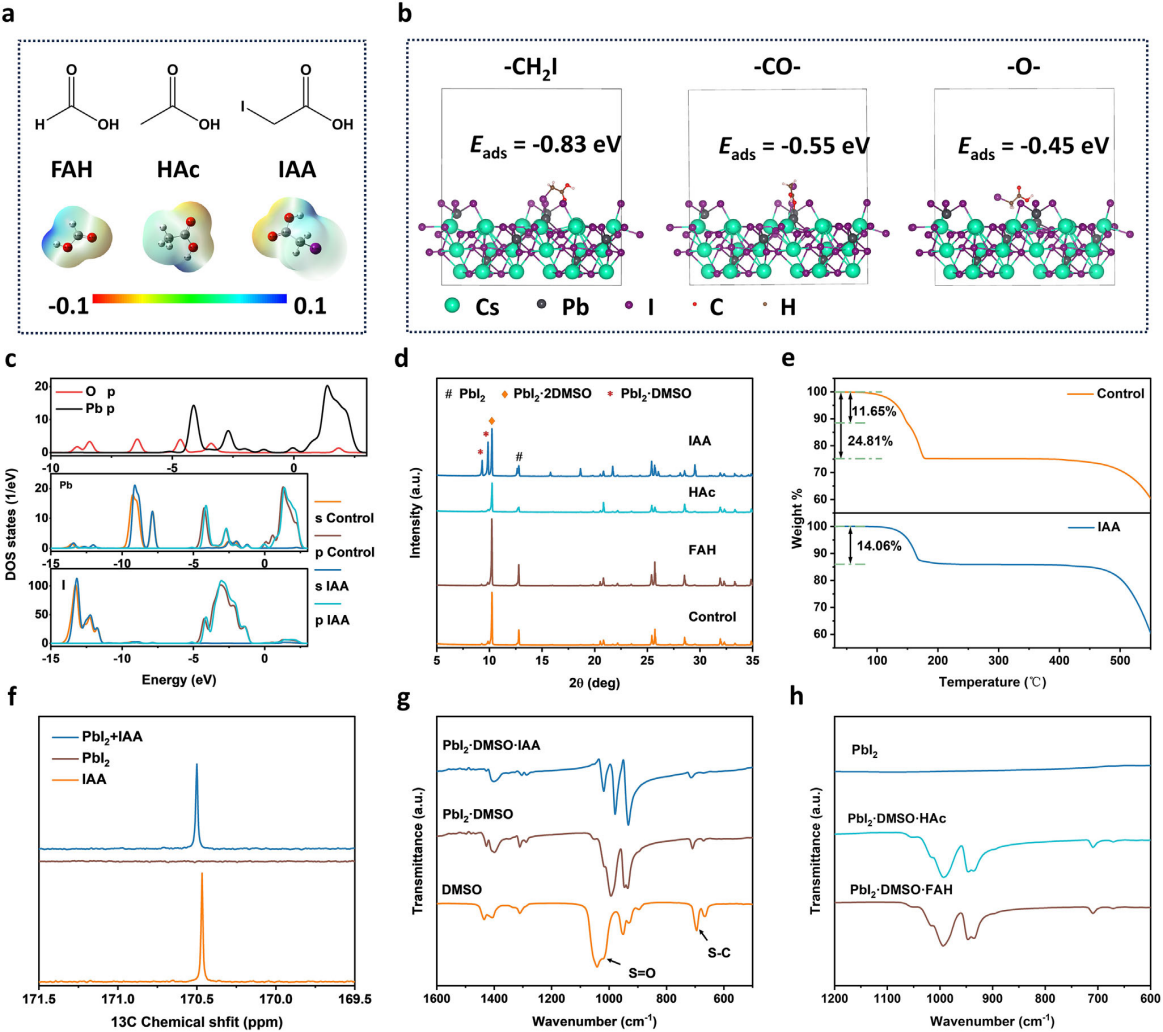
(a) Molecular structure diagrams and electrostatic potential surface (EPS) images of FAH, HAc, and IAA. b) DFT‐calculated adsorption energies of IAA adsorbed on the Cs_4_PbI_6_ (001) surface via ‐CH_2_I, ‐CO‐, and ‐O‐. (c) Calculated density of states (DOS) for pristine and IAA‐adsorbed Cs_4_PbI_6_. (d) XRD patterns of PbI_2_‐DMSO powders. (e) Thermogravimetric analysis (TGA) of PbI_2_+DMSO and PbI_2_+DMSO+IAA powders. (f) ^13^C NMR spectroscopy of IAA, PbI_2_, and PbI_2_+IAA solutions. FTIR spectra of (g) DMSO, PbI_2_+DMSO, and PbI_2_+DMSO+IAA and (h) PbI_2_+DMSO+FAH, PbI_2_+DMSO+HAc, and PbI_2_.

To understand how the ‐CH_2_I group influences the conformational dynamics of IAA and its subsequent coordination with Pb^2+^, we systematically analyzed the electrostatic potential surface (EPS) distributions using density functional theory (DFT) (Figure 1a). The distinct electronic characteristics of the substituents dictate the molecular interaction profiles. Notably, IAA exhibits a unique bipolar EPS distribution, the carboxyl group displays a concentrated negative potential, indicative of strong Pb^2+^ coordination capability. Furthermore, the electron‐withdrawing ‐I group polarizes the O‐H bond, facilitating deprotonation and strengthening the resultant Pb‐O bond. This molecular architecture enables a quasi‐bidentate coordination mode. The terminal iodine atom provides secondary weak interaction sites, allowing IAA to form stronger and more stable adsorption complexes on the crystal surface. Additionally, the lability of the C‐I bond facilitates heterolytic cleavage, releasing I^−^ to compensate for V_I_. The large atomic radius and distinct charge distribution of iodine create pronounced spatial and electronic shielding, effectively preventing solvent molecules (DMSO/DMF) from re‐coordinating with Pb^2+^. In contrast, FAH and HAc exhibit monofunctional characteristics, FAH favors strong but monodentate coordination, whereas the delocalized polarity of HAc results in weaker, non‐specific interactions with the crystal surface.

To further elucidate the preferential adsorption configurations, we calculated the binding energies of IAA on the Cs_4_PbI_6_ (001) surface (Figure 1b). The research results show a clear hierarchy of energy, with adsorption mediated by the ‐CH_2_I group being the most favorable (−0.83 eV), significantly stronger than coordination via carbonyl oxygen (−0.55 eV) or hydroxyl group (−0.45 eV). This underscores the strong affinity of the ‐CH_2_I moiety for the I‐rich surface, likely governed by van der Waals forces and halogen‐bond‐like interactions, which together form a sterically protective interfacial barrier. Notably, IAA exhibits a substantially higher adsorption energy compared to FAH (−0.31 eV) and HAc (−0.32 eV) (Figure S1), confirming its superior surface‐binding capability. Projected density of states (PDOS) analysis reveals significant orbital hybridization between the carboxyl O 2p states and Pb 6p states (Figure 1c; Figure S2), providing electronic evidence for strong coordinative bond formation. Furthermore, the suppression of mid‐gap trap states near the Fermi level, which are typically associated with undercoordinated Pb^2+^, thus provides electronic evidence of effective trap passivation. Bader charge analysis indicates a net charge transfer of 0.38*e* ^−1^ from IAA to the Cs_4_PbI_6_ surface (Figure S3), demonstrating robust electronic coupling. Critically, IAA adsorption significantly increases the formation energies of key intrinsic point defects, including V_Cs_ (from 3.47 to 3.63 eV), V_Pb_ (from 2.43 to 2.87 eV), and most notably V_I_ (from 0.88 to 1.64 eV) (Figure S4). This dramatic increase in defect formation energies reflects IAA's ability to stabilize the perovskite lattice and suppress intrinsic defect generation, aligning with the experimentally observed iodine‐compensation behavior arising from IAA decomposition, further validating the proposed iodine compensation mechanism in the trifunctional kinetic regulation.

To elucidate molecular‐level interactions governing precursor coordination, we synthesized single crystals of PbI_2_‐solvent adducts derived from DMSO and DMF solutions (Figure 1d) [31, 32]. The XRD patterns of the adduct from the pristine PbI_2_‐DMSO solution were dominated by sharp peaks characteristic of the PbI_2_·2DMSO, accompanied by minor peaks corresponding to PbI_2_·DMSO. This indicates a thermodynamic preference for a 2:1 (DMSO: PbI_2_) coordination stoichiometry in the absence of additives. Adducts prepared with FAH or HAc exhibited profiles similar to the control, suggesting limited capacity to disrupt the stable PbI_2_·2DMSO structure. In contrast, the IAA‐containing system displayed a markedly distinct pattern characterized by the coexistence of PbI_2_·DMSO and PbI_2_·2DMSO phases, with a concomitant suppression of the PbI_2_·2DMSO peaks. This demonstrates the efficacy of IAA in competitively coordinating with Pb^2+^, thereby inhibiting the formation of a single, highly ordered adduct and promoting a more disordered intermediate state. A more pronounced effect was observed in the weaker coordinating solvent DMF (Figure S5) [33]. While additives FAH and HAc still yielded adducts containing PbI_2_·DMF complexes, the product from the IAA‐DMF solution was dominated by diffraction peaks of pristine PbI_2_, with PbI_2_·DMF peaks being significantly suppressed. This indicates that IAA effectively displaces DMF molecules from the PbI_2_ coordination sphere, directly binding to PbI_2_ and preventing solvent‐dominated adduct crystallization. Thermogravimetric analysis (TGA) provided quantitative insights into thermal stability that complemented the XRD findings (Figure 1e) [31]. The control PbI_2_‐DMSO adduct exhibited two distinct weight loss steps, initiating at ∼58°C and ∼148°C, corresponding to the stepwise desorption and evaporation of DMSO molecules from distinct coordination environments. The calculated molar ratios of DMSO to PbI_2_ derived from these losses were approximately 1:1.29 and 1:1.95, quantitatively confirming the predominance of the 1:2 (PbI_2_·2DMSO) adduct alongside a minor 1:1 (PbI_2_·DMSO) component. Conversely, the IAA‐modified adduct displayed a single major weight loss step, corresponding to a DMSO: PbI_2_ molar ratio of ∼1:1.03. This TGA data unequivocally demonstrates that IAA incorporation alters the adduct's thermal behavior and composition, shifting the equilibrium toward a dominant 1:1 coordinated PbI_2_·DMSO species, in perfect alignment with the XRD evidence regarding the suppression of the ordered PbI_2_·2DMSO phase.

Direct spectroscopic evidence for the solution‐state interaction between IAA and lead species was obtained via ^13^C NMR spectroscopy (Figure 1f). The carboxyl carbon resonance of pristine IAA was appeared at 170.47 ppm. Upon the addition of PbI_2_, this peak exhibited a distinct downfield shift to 170.50 ppm, confirming the coordination of the IAA carboxylate group with PbI_2_ to form an IAA‐Pb complex within the precursor solution. This coordination event represents a crucial step in the proposed mechanism, as it competitively disrupts the dominant PbI_2_‐DMSO solvation structure. Further evidence for competitive ligand exchange in the precursor solution was obtained from FT‐IR spectroscopy of the PbI_2_‐DMSO adducts (Figure 1g,h; Figure S6) [34]. The formation of the PbI_2_‐DMSO adduct was corroborated by a red‐shift of the S = O stretch (from 1019.7 to 934.8 cm^−1^) and a blueshift of the C‐S vibration (from 695.2 cm^−1^ to 709.7 cm^−1^). The introduction of IAA induced further changes, with the C‐S vibration further blueshift to 714.5 cm^−1^, the emergence of a new peak at 979.2 cm^−1^ (attributed to a hydrogen‐bonded S = O···H‐O vibration), and the coexistence of features for both coordinated (933.4 cm^−1^) and free DMSO (1019.2 cm^−1^). These spectral features clearly indicate that IAA perturbs the PbI_2_‐DMSO coordination equilibrium through competitive complexation and hydrogen‐bond formation.

**FIGURE 2:**
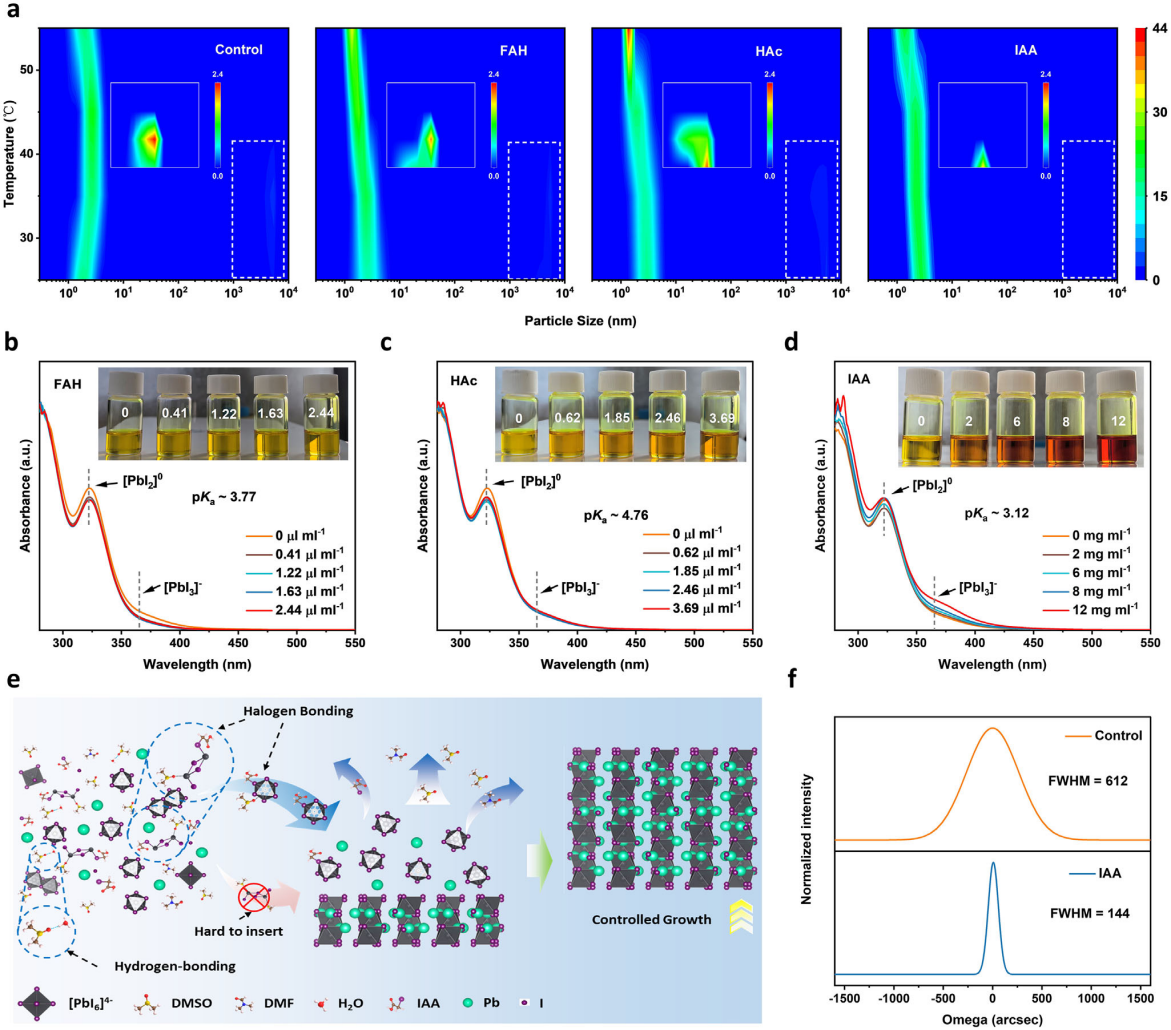
(a) Temperature‐dependent DLS curves of Cs_4_PbI_6_, Cs_4_PbI_6_+FAH, Cs_4_PbI_6_+HAc, and Cs_4_PbI_6_+IAA. The inset is a magnified view of the large particle size range. UV–vis absorption spectra representing the evolution of characteristic peak intensity while continuously adding (b) FAH, (c) HAc, and (d) IAA. (e) Schematic diagram of crystallization kinetics control in Cs_4_PbI_6_ crystal growth. (f) XRD rocking curve of the (223) diffraction of Cs_4_PbI_6_ crystal.

The evolution of precursor solutions, which is critical for crystal growth, was monitored via temperature‐dependent dynamic light scattering (DLS) from 25°C to 55°C (Figure 2a; Figure S7) [35]. At 25°C, all precursors exhibited a bimodal size distribution, with the primary peak (0.7–8.3 nm) for solvated [PbI_6_] [4^−^] octahedral clusters and a broad distribution (10^3^–10^4^ nm) for large‐scale aggregation. Upon heating, the control group displayed non‐monotonic changes, with macroaggregates persisting until 45°C. The introduction of FAH or HAc altered this behavior, while the population of small clusters decreased monotonically with temperature, suggesting moderate stabilization, but macroaggregates persisted until 45°C, reflecting their limited efficacy. In contrast, the IAA‐containing precursor exhibited superior behavior. Although small aggregates decreased directly with temperature, the key distinction was the complete dissolution of large aggregates by 35°C, a full 10°C earlier than with FAH or HAc. Achieving a monodispersed state at this lower temperature facilitates a more uniform nucleation process and layer‐by‐layer growth.

Further insight into the precursor chemistry was obtained via UV–vis absorption spectroscopy (Figure 2b–d) [36]. The absorption peaks at 323 and 365 nm were attributed to lead‐iodide complexes [PbI_2_]^0^ and [PbI_3_]^−^, respectively. The addition of FAH (p*K*_a_∼3.77) and HAc (p*K*_a_∼4.76) induced complex, non‐monotonic changes in the [PbI_3_]^−^ absorption peak, a phenomenon primarily governed by the pH‐dependent iodine disproportionation equilibrium [37, 38, 39]. At higher concentrations, the acetate anion engages in weak coordination with solution species, potentially fine‐tuning solubility equilibria or facilitating the oxidation of I^−^ to I_2_. In contrast, while the high acidity of IAA (p*K*_a_∼3.12) would be expected to suppress the iodine disproportionation equilibrium via a pH effect, this influence is overridden by the direct release of I^−^ through heterolytic cleavage of the C‐I bond. The exogenous I^−^ actively participates in the disproportionation equilibrium, driving it toward the generation of [PbI_3_]^−^. This results in a significant net increase in the concentration of [PbI_3_]^−^ complexes, manifested as a progressively intensifying absorption peak and a deepening color of the solution. Consequently, IAA functions not merely as a pH regulator but as an active iodine‐compensating agent. After 48 h of aging, the FAH and HAc‐modified precursor solutions exhibited only a marginal increase in the 365 nm absorption band (Figure S8). In contrast, the IAA‐modified solution displayed substantially stronger absorption at 365 nm, consistent with a higher concentration of [PbI_3_]^−^ complexes and sustained I^−^ release. Variable‐temperature UV–vis spectroscopy was employed to monitor the evolution of I‐related species under conditions relevant to crystal growth (Figure S9) [40]. As the temperature increased, the absorption peaks corresponding to I species were progressively enhanced in both the control and IAA‐doped systems. Since the iodine disproportionation reaction is endothermic, increasing the temperature shifts the equilibrium toward the products, thereby promoting the formation of the strongly absorbing [PbI_3_]^−^. Acting as an intrinsic I^−^ source, IAA uniquely leverages thermal energy not only to drive the inherent equilibrium but also to activate its function as an iodine‐compensation reservoir. This synergistic, thermally activated iodine release mechanism ensures a rich supply of reactive iodine species precisely during the heated crystal growth phase, effectively compensating for potential iodine vacancies and facilitating the formation of high‐quality, stoichiometrically balanced Cs_4_PbI_6_ single crystals.

Based on these observations, we propose a coherent molecular mechanism (Figure 2e). In solution, IAA undergoes partial deprotonation, generating a ‐COO^−^ with superior affinity for PbI_2_ compared to DMSO. This carboxylate chelates PbI_2_, displacing a portion of the coordinated DMSO molecules. Concurrently, the cleavage of the C‐I bond serves as a dynamic iodine reservoir to promptly provide iodine compensation. The proton (H^+^) released from IAA is captured by trace water to form hydronium ions (H_2_O·H^+^), which subsequently engage the liberated DMSO molecules via an O = S···H‐O hydrogen‐bonding network. This synergy of competitive coordination and hydrogen bonding effectively modulates the precursor's colloidal stability and nucleation kinetics.

Building upon this mechanistic analysis, we devised a strategy for growing Cs_4_PbI_6_ single crystals utilizing the inverse temperature crystallization (ITC) method [22]. As illustrated in Figure S10, after 12 h of stirring, the precursor solution gradually transformed from pale yellow to dark reddish‐brown, which can be attributed to the cleavage of the C‐I bond in IAA and the subsequent release of I^−^ into the solution. Following filtration, the solution was maintained at 35°C for another 24 h to minimize the formation of large aggregated particles, during which I^−^ continue to be released. This phenomenon aligns with the spectral evolution revealed by UV–vis analysis. With the increase in temperature, nucleation proceeded from single sites, gradually yielding large Cs_4_PbI_6_ single crystals with well‐defined facets and low defects densities. Significant differences in crystal morphology were observed by the naked eye when FAH, HAc, and IAA are used as functional additives (Figure S11). While the crystals obtained in the control group were of moderate size, the additive‐assisted groups, particularly the IAA group, yielded single crystals with significantly larger size and higher visual transparency. This indicates a notable improvement in crystal quality and a reduction in optical scattering centers. Statistical analysis of crystal sizes from multiple batches further corroborates this finding, with the IAA group consistently yielding the largest crystals.

To unequivocally confirm phase purity and structural identity, powder X‐ray diffraction (XRD) was performed. The XRD patterns (Figure S12) for crystals from all groups exclusively exhibited characteristic diffraction peaks at 27.69°, 39.51°, and 48.88°, corresponding to the standard structure of Cs_4_PbI_6_ (ICSD#25124) [41]. Notably, the absence of detectable peaks from impurities, such as the photoactive CsPbI_3_ phase or PbI_2_, confirms that the carboxylic acid additives did not alter the final crystalline phase. We further assessed the crystallinity by measuring the X‐ray rocking curves of the (223) planes (Figure 2f). The IAA‐regulated single crystals exhibited superior crystal quality with a full‐width at half‐maximum (FWHM) of 144 arcsec, a significant improvement over the 612 arcsec observed for the control group. Additionally, the bandgap of the crystal powder was determined to be 3.65 eV via Tauc plot analysis (Figure S13). To verify the chemical composition and the proposed steric mechanism, NMR and FTIR analyses were conducted (Figure S14). These results confirm the absence of IAA residues in the final lattice. Notably, NMR integration and elemental analysis reveal a significant reduction in residual solvent content (DMSO), validating that IAA effectively inhibits solvent incorporation via steric hindrance without entering the crystal lattice. The extended lifetimes (τ_1_, τ_2_, τ_3_) further demonstrate effective defect passivation and suppressed non‐radiative recombination, corroborating the substantial enhancement in overall crystal quality indicated by the XRD results (Figure S15).

**FIGURE 3:**
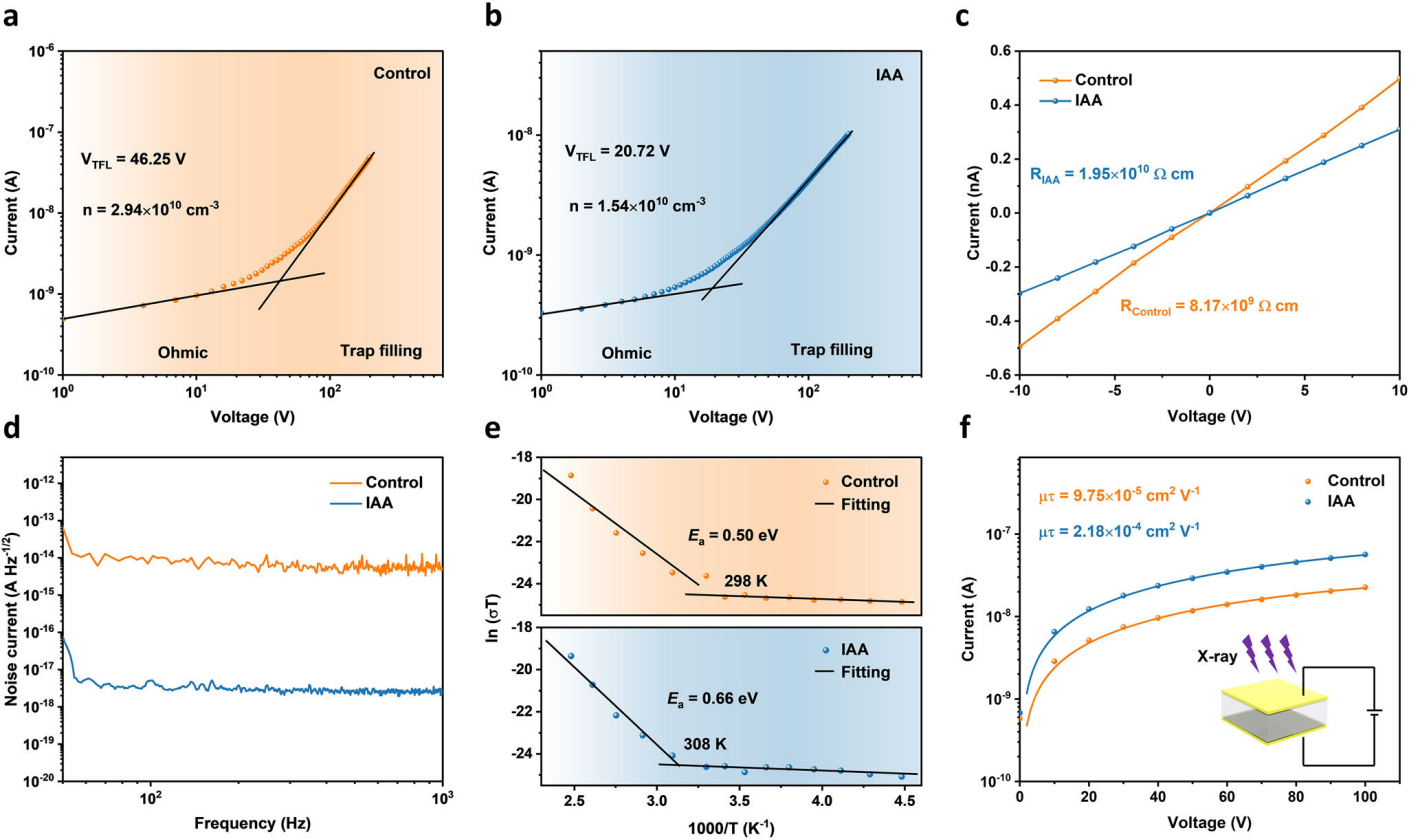
Space‐charge‐limited current (SCLC) characteristics of the (a) Control and (b) IAA devices. (c) Dark current‐voltage (*I–V*) curves. (d) Noise current of the Control and IAA devices (The bias voltage is 5 V). (e) Temperature‐dependent electrical conductivity plots of the Control and IAA devices. (f) Carrier mobility‐lifetime (µτ) product. The inset is a schematic diagram of the device structure.

To quantitatively assess the impact of IAA regulation on the electrical performance of Cs_4_PbI_6_ SCs, we conducted a series of electrical measurements on Au/Cs_4_PbI_6_/Au symmetric devices. The trap density (n) was first quantified using the space‐charge‐limited current (SCLC) method (Equation S1) [42]. As shown in Figure 3a,b, the dark current‐voltage curves for both the control group and the IAA‐regulated devices exhibit a clear transition from the ohmic region to the trap‐filled region at the trap‐filled limit voltage (*V*_TFL_). Notably, the *V*_TFL_ value significantly decreased from 46.25 V for the control device to 20.72 V for the IAA‐regulated device. Consequently, the calculated trap density decreased from 2.94 × 10^10^ cm^−3^ to 1.54 × 10^10^ cm^−3^. This reduction of trap density by more than 47.62% provide direct evidence that the kinetic iodine‐compensation of IAA effectively passivates deep‐level traps. Concomitant with the decrease in trap states, the crystal resistivity calculated from the dark *I–V* characteristics (Figure 3c) increased from 8.17 × 10^9^ Ω cm to 1.95 × 10^10^ Ω cm. The substantial increase in resistivity is attributed to the suppression of ion migration and the reduction of defect‐related charge leakage pathways, thereby minimizing the dark current under bias. Since device noise dictates the minimum detectable signal, we measured the noise current of the photodetectors across a range of frequencies [43, 44]. Owing to the significant reduction in defect density and the concomitant increase in resistivity, the noise current of the IAA‐regulated single‐crystal devices is markedly lower than that of the control devices (Figure 3d).

To further investigate the suppression mechanism of ion migration, we conducted temperature‐dependent conductivity measurements. By analyzing the variation of conductivity (σ) with temperature (T) and fitting the data to the Arrhenius equation (Figure 3e) [45], we determined that the IAA‐regulated devices exhibit a significantly higher activation energy (*E*_a_ = 0.66 eV), compared to the control devices (*E*_a_ = 0.50 eV). This elevated activation energy indicates that ion migration must overcome a higher energy barrier, confirming that the IAA additive can effectively stabilizes the lattice structure and suppress the ion migration phenomena typically observed in perovskite devices under electrical stress. Furthermore, by analyzing the photocurrent versus applied bias curves using the Hecht equation (Figure 3f) [46], the carrier mobility‐lifetime product (µτ) was calculated to increase significantly from 9.75 × 10^−5^ cm^2^ V^−1^ for the control devices to 2.18 × 10^−4^ cm^2^ V^−1^ for the IAA‐regulated devices. This enhancement in charge transport properties is directly attributed to the reduction of trap‐assisted recombination centers and the suppression of ion migration, both of which synergistically allow photogenerated carriers to travel longer distances before being trapped or recombined.

**FIGURE 4:**
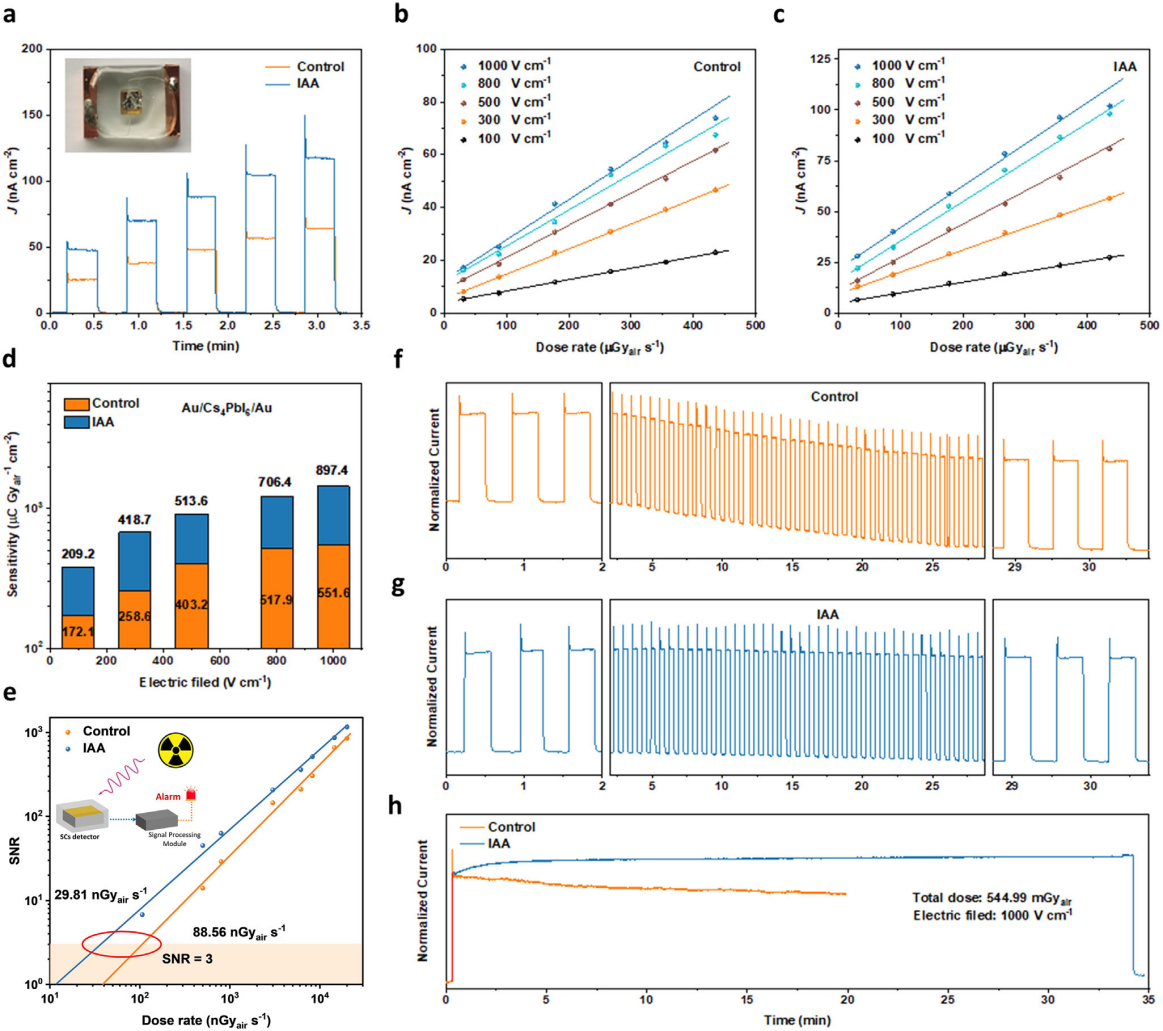
(a) Photocurrent response of the SCs detector under an 80 keV X‐ray source at an applied electric field of 1000 V cm^−1^. The inset is the photographs of the single crystal detector. Photocurrent response of (b) the control and (c) IAA device under varying electric fields (100, 300, 500, 800, and 1000 V cm^−1^). (d) Bias‐dependent detector sensitivity. (e) Dose rate‐dependent SNR of the Cs_4_PbI_6_ SCs detector. Inset is the schematic diagram of SCs Detector Monitoring. The X‐ray on/off cycles response of (f) control and (g) IAA under a constant bias of 1000 V cm^−1^. (h) Long‐term irradiation stability test.

To evaluate the practical potential of Cs_4_PbI_6_ single crystals for X‐ray detection, we systematically characterized their key performance metrics. Based on the NIST database, the mass attenuation coefficient of Cs_4_PbI_6_ was calculated [11, 47, 48]. Owing to the high atomic numbers of its constituent elements (Cs, Pb, and I), Cs_4_PbI_6_ exhibits a large X‐ray absorption cross‐section. Calculations indicate that a 1‐mm‐thick single crystal can absorb approximately 77.19% of incident X‐ray photons at an energy of 80 keV (Figures S16 and S17). This high attenuation efficiency ensures effective photon capture, providing a solid foundation for high‐sensitivity detection. Furthermore, we systematically evaluated the actual X‐ray detection performance of Cs_4_PbI_6_ SCs devices. As shown in Figure 4a, the device adopts a symmetric design, with the crystal sandwiched between two Au electrodes to achieve direct charge collection [49]. Under a fixed electric field strength of 1000 V cm^−1^, we measured the current response of the IAA‐regulated devices under varying dose rates (80 keV X‐ray irradiation). The current spike observed in the device is typically attributed to the synergistic effect of trap filling and ion migration. Upon X‐ray turn‐on, a large number of photogenerated carriers are rapidly captured by vacant deep‐level traps, forming a transient filling current that manifests as an initial current overshoot. Once these traps become saturated, the current relaxes to a steady‐state level. Concurrently, under the applied electric field, mobile ions within the perovskite migrate, altering the local electric field distribution. This ionic motion superimposes on the trap‐filling process, further enhancing the transient response by modulating the interfacial electric fields [50, 51]. Figure 4b,c demonstrates an excellent linear relationship between photocurrent and dose rate. From the photocurrent‐dose rate relationship, the sensitivity of the control device was calculated to be 551.6 µC Gy_air_^−1^ cm^−2^, whereas the sensitivity of the IAA‐regulated device was significantly enhanced to 897.4 µC Gy_air_^−1^ cm^−2^ (Figure 4d; Figure S18). This performance improvement of over 60% is directly attributed to the superior charge transport properties resulting from IAA‐mediated crystal growth. By performing a linear fit of the dose rate dependent SNR and extrapolating to an SNR of 3, the detection limit was determined to be 29.81 nGy_air_ s^−1^ (Figure 4e). Through periodic X‐ray on‐off cycle tests, we investigated the operational stability of the single‐crystal devices under dynamic irradiation conditions. While the control device exhibited significant signal drift (Figure 4f), the IAA‐modified device showed highly stable and repeatable responses, with negligible signal attenuation after multiple cycles (Figure 4g). This enhanced cyclability is attributed to suppressed ion migration and reduced charge trapping in the IAA‐modified crystals. In continuous irradiation tests (Figure 4h), the superiority of the IAA‐modified device was even more evident. The control device suffered from severe signal drift due to defect migration and field‐induced polarization, whereas the IAA‐modified device maintained a stable photocurrent output, demonstrating excellent resistance to operational fatigue. Long‐term monitoring of the dark current baseline (Figure S19) quantitatively confirmed the improvement in stability. The baseline drifted of the control devices was 6.28 × 10^−5^ nA cm^−1^ s^−1^ V^−1^, while the IAA‐modified device showed only 1.43 × 10^−5^ nA cm^−1^ s^−1^ V^−1^. This verifies the effectiveness of IAA in constructing a more stable and reliable lattice structure, minimizing noise, and ensuring long‐term accurate signal readout.

## 3 Conclusions

In conclusion, we have demonstrated a rational molecular design strategy employing IAA as a trifunctional regulator to govern the crystallization of 0D Cs_4_PbI_6_ single crystals. By simultaneously addressing the challenges of deep‐level traps, iodine vacancies, and solvent incorporation, this strategy yields high‐quality single crystals with ultralow defect densities. The resulting detectors exhibit excellent electronic properties, combining high sensitivity (897.4 µC Gy_air_^−1^ cm^−2^), an ultralow detection limit (106.5 nGy_air_ s^−1^), and exceptional operational stability. This work not only provides a fundamental understanding of defect physics in solution‐processed semiconductors but also opens a new avenue for developing next‐generation high‐performance optoelectronic materials through kinetic regulation.

## Author Contributions

H.Z. and Z.W. contributed equally to this work. H.Z. and J.Y. conceived the main idea of this work. H.Z. was responsible for investigation, visualization, data organization, and drafting the original manuscript. J.Y. supervised the project and process. H.Z., Z.W., Z.W., and C.W. fabricated devices and performed photovoltaic measurements. Z.L., H.X., Y.M., and Y.L. contributed to or assisted with subsequent experimental characterizations and data analysis. J.Y. and X.P. revised the manuscript. All authors discussed the results and commented on the manuscript.

## Conflicts of Interest

The authors declare no conflicts of interest.

## Supporting information




**Supporting File**: advs75517‐sup‐0001‐SuppMat.docx.

## Data Availability

The data that support the findings of this study are available from the corresponding author upon reasonable request.
