# Supplementary material for: Trifunctional Kinetic Regulation Enables Low‐Defect Solution Grown Single Crystals for X‐Ray Detection
Source: Adv Sci (Weinh). 2026 May 30;13(42):e75517. doi: 10.1002/advs.75517 (PMC13335657; doi:10.1002/advs.75517)
Supplement: Supplementary file 1 — Supporting File: advs75517‐sup‐0001‐SuppMat.docx. [file ADVS-13-e75517-s001.docx]

Supporting Information

**Trifunctional Kinetic Regulation Enables Low-Defect Solution Grown Single Crystals for X-ray Detection**

*Hui Zhang* ^&^*, Zihan Wang* ^&^*, Zhenyu Wang, Changmao Wan, Zheng Liang, Huifen Xu, Yuanbo Ma, Yupeng Liu, Xu Pan, and Jiajiu Ye ^*^*

& These authors contributed equally to this work.

H. Zhang, Z. Wang, Z. Wang, C. Wan, Z. Liang, H. Xu, Y. Ma, Y. Liu, X. Pan, J. Ye

Institute of Solid-State Physics

Hefei Institutes of Physical Science

Chinese Academy of Science

Hefei 230031, China

E-mail: [yejj@issp.ac.cn](mailto:yejj@issp.ac.cn)

H. Zhang, C. Wan, Y. Ma, Y. Liu

University of Science and Technology of China

Hefei 230026, China

**Keywords:** Trifunctional, Kinetic Regulation, Single Crystals, Cs_4_PbI_6_, X-ray Detection

**Experimental Section**

*Materials*: Cesium iodide (CsI, 99.9%, metals basis), Iodoacetic acid (IAA, 98%) Formic acid (FAH, 99%) were purchased from Innochem. lead (II) iodide (PbI_2_, 99%,) was purchased from Adamas. Acetic acid (HAc, 99.5%) was purchased from TCI. N, N-dimethylformamide (DMF, AR, ≥ 99.5%) and dimethylsulfoxide (DMSO, anhydrous, 99.9%, Water ≤ 50 ppm) were purchased from Aladdin. All materials can be used directly without further purification.

*Cs_4_PbI_6_ single crystals (SCs) growth*: Cs_4_PbI_6_ single crystals were grown by inverse temperature crystallization (ITC) using the solution method. CsI and PbI_2_ were weighed in a stoichiometric ratio of 4:1 and dissolved in a mixed solvent (*V*_DMF_: *V*_DMSO_ = 4:1) to form a precursor solution with a concentration of 0.3 M. After the mixed solution was stirred at room temperature for 12 h, it was filtered through a PTFE filter with a pore size of 0.45 μm filter to obtain a clear solution. Place the solution on a 35°C hot plate and keep it there for 24 hours. Then, heat it to 80°C at a rate of 5°C/day using programmed temperature control, and maintain this temperature for several days. Finally, high-quality Cs_4_PbI_6_ single crystals were obtained from the solution. After blotting the solution with absorbent paper, immerse the single crystals in a beaker containing isopropanol for ultrasonic cleaning. Wipe the crystal surfaces with a non-woven cloth moistened with isopropanol to thoroughly remove microcrystals, then perform ultrasonic cleaning with isopropanol again. After completely cleaning the crystal surfaces, place them in a 50°C vacuum oven to dry overnight.

*PbI_2_-DMSO single crystals (SCs) growth*: PbI_2_-DMSO single crystals were prepared by the vapor diffusion method. Specifically, 4.61 g of PbI_2_ was dissolved in 10 mL of DMSO with stirring for 6 hours until complete dissolution. Solutions were prepared for the control group and experimental groups containing FAH, HAC, and IAA, respectively. The resulting solution was filtered using a 0.22 μm PTFE filter into a 25 mL beaker. A 200 mL beaker was charged with 40 mL of ethyl acetate as the diffusion agent. The beaker containing the solution was placed, uncovered, inside the larger 200 mL beaker. The larger beaker was sealed with plastic wrap and kept at room temperature to allow for slow vapor diffusion and crystal formation. The obtained crystals were carefully collected, and their surfaces were gently wiped clean. Finally, the crystals were stored overnight in a vacuum desiccator to remove residual solvent.

*PbI_2_-DMF single crystals (SCs) growth*: PbI_2_-DMF SCs adopts the same synthesis method as PbI_2_-DMSO SCs, only requiring DMSO to be replaced with DMF, while all other operations remain unchanged.

*Device Fabrication*: For preparation of the Au/Cs_4_PbI_6_/Au device, the Au electrode was evaporated on the surface of the Cs_4_PbI_6_ SCs by vacuum evaporation method, with a thickness of ~80 nm. It is worth noting that all the devices are symmetrical plane electrodes.

*Computational details*: All the DFT calculations were conducted based on the Vienna Ab initio Simulation Package (VASP).^[1, 2]^ The exchange-correlation potential was described by the Perdew-Burke-Ernzerhof (PBE) generalized gradient approach (GGA).^[3]^ The electron-ion interactions were accounted by the projector augmented wave (PAW).^[4]^ All DFT calculations were performed with a cut-off energy of 400 eV, and the 1×1×1 Gamma centered Monkhorst-Pack grids k-points were selected to sample the Brillouin zone integration. The energy and force convergence criteria of the self-consistent iteration were set to 10^-5^ eV and 0.05 eV Å^-1^, respectively. DFT-D3 method was used to describe van der Waals (vdW) interactions.^[5]^

*Electrical properties and device performance* *measurements*: All tests are conducted in a closed dark lead chamber at room temperature to reduce the interference of complex signals in the test environment. Bias voltage and current signal acquisition is provided using a Keithley 6517b electrometer. The X-ray source model involved in this work is the commercially available Hamamatsu L11831 (tungsten target, maximum tube voltage 90 kV, maximum tube current 200 μA). The X-ray dose rate was calibrated with a Radcal Accu-Dose+ 10 × 6 - 180 dosimeter.

*Characterization*: The UV-vis absorption spectrum was analyzed using Hitachi U3900H, with a test range of 280-900 nm. Powder XRD was carried out using an XRD diffractometer of Rigaku Smart Lab 9 kW, which recorded the diffraction signal of Cu Kα in the range of 5-80°.

**Space-charge-limited current (SCLC).** The trap density is calculated by measuring the dark current-voltage (I-V) curves of the SCs. The average trap density is calculated using the following formula:

$$\begin{aligned} \text{n}_{\text{trap}}\text{=}\frac{\text{2ε}\text{ε}_{\text{0}}\text{V}_{\text{TFL}}}{\text{q}\text{L}^{\text{2}}}\#\left( \text{1} \right) \end{aligned}$$

where *ε* is the dielectric constant (*ε*_Control_ = 5.76, *ε*_IAA_ = 6.73, Test frequency is 10 kHz), and *ε*_0_ the vacuum permittivity, respectively. *q* is the elementary charge, *L* the thickness of the SC, and *V*_TFL_ the trap-filled limit voltage.

**Mobility-lifetime products (μτ).** The μτ product was calculated according to the simplified single-carrier Hecht equation:

$$\begin{aligned} \text{I}\text{ = }\frac{\text{I}_{\text{0}}\text{μτV}}{\text{L}^{\text{2}}}\frac{\text{1-exp(-}\frac{\text{L}^{\text{2}}}{\text{μτV}}\text{)}}{\text{1+}\frac{\text{L}}{\text{V}}\frac{\text{s}}{\text{μ}}}\#\left( \text{2} \right) \end{aligned}$$

where *I*_0_ is the saturated photocurrent, *L* is the thickness of crystal and *V* is the applied bias.

**Signal to Noise Ratio (SNR).** The SNR was calculated from equation:

$$\begin{aligned} \text{SNR }\text{ = }\frac{\text{I}_{\text{signal}}}{\text{I}_{\text{noise}}}\text{ = }\frac{\text{I}_{\text{p}}\text{-}\text{I}_{\text{d}}}{\sqrt{\frac{\text{1}}{\text{N}}\sum_{\text{i}}^{\text{N}} \left( \text{I}_{\text{i}}\text{-}\text{I}_{\text{p}} \right)^{\text{2}}}}\#\left( \text{3} \right) \end{aligned}$$

where *I*_p_ is the average photocurrent density, *I*_d_ is the average dark-current density and *I_i_* is the instantaneous value of the photocurrent.

**Sensitivity (S).** X-ray sensitivity (S) of the detectors can be calculated by the following equation:

$$\begin{aligned} \text{S}\text{ = }\frac{\text{I}_{\text{p}}\text{-}\text{I}_{\text{d}}}{\text{DA}}\#\left( \text{4} \right) \end{aligned}$$

where *D* is the dose rate of incident X-ray radiation, and *A* is the active area of the detector.

**Baseline drift (*I*_drift_).** The dark current drift (*I*_drift_) was calculated from equation:

$$\begin{aligned} \text{I}_{\text{drift }}\text{= }\frac{\text{I}_{\text{t}}\text{-}\text{I}_{\text{0}}}{\text{EAt}}\#\left( \text{5} \right) \end{aligned}$$

where *I*_t_ is the current at time t, *I*_0_ is the origin current immediately after stability, E is the electric field intensity, and *A* is the active area of the detector.

**X-Ray Attenuation Coefficients (μ).** The X-ray attenuation coefficient is a basic quantity for calculating the penetration of X-ray photons in biological, shielding, and other materials. The relationship between attenuation coefficient and material thickness can be calculated according to the formula:

$$\begin{aligned} \text{μd}\text{ = }\ln\left( \frac{\text{I}_{\text{0}}}{\text{I}} \right)\#\left( \text{6} \right) \end{aligned}$$

where *I*_0_ is incident intensity, *I* is penetration intensity, *μ* is attenuation coefficient, and *d* is the thickness of the detector.


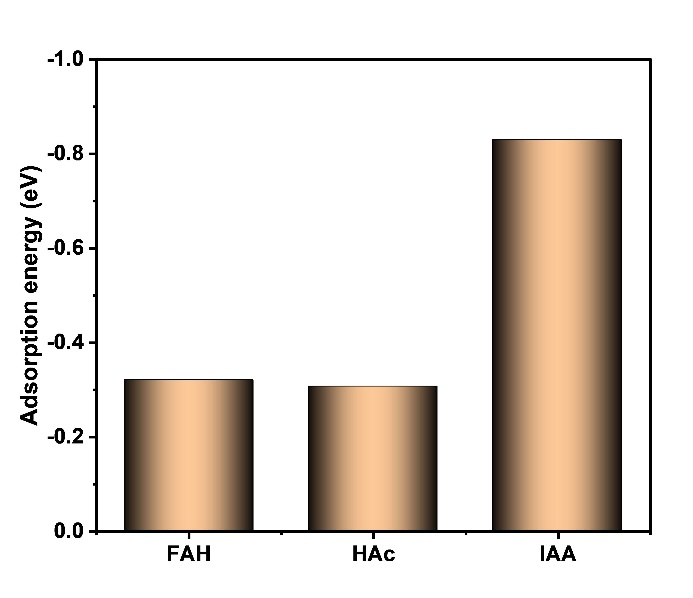


**Figure S1.** DFT-calculated adsorption energy comparison of FAH, HAc, and IAA on the Cs_4_PbI_6_ (001) surface.


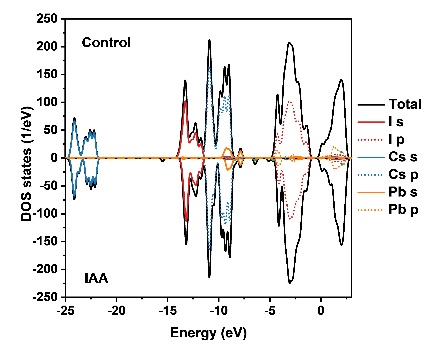


**Figure S2.** Calculated density of states (DOS) for pristine and IAA-adsorbed Cs_4_PbI_6_.

**
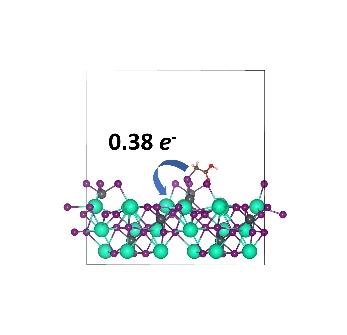
**

**Figure S3.** Calculate the average Bader charge between IAA and Cs_4_PbI_6_.

**
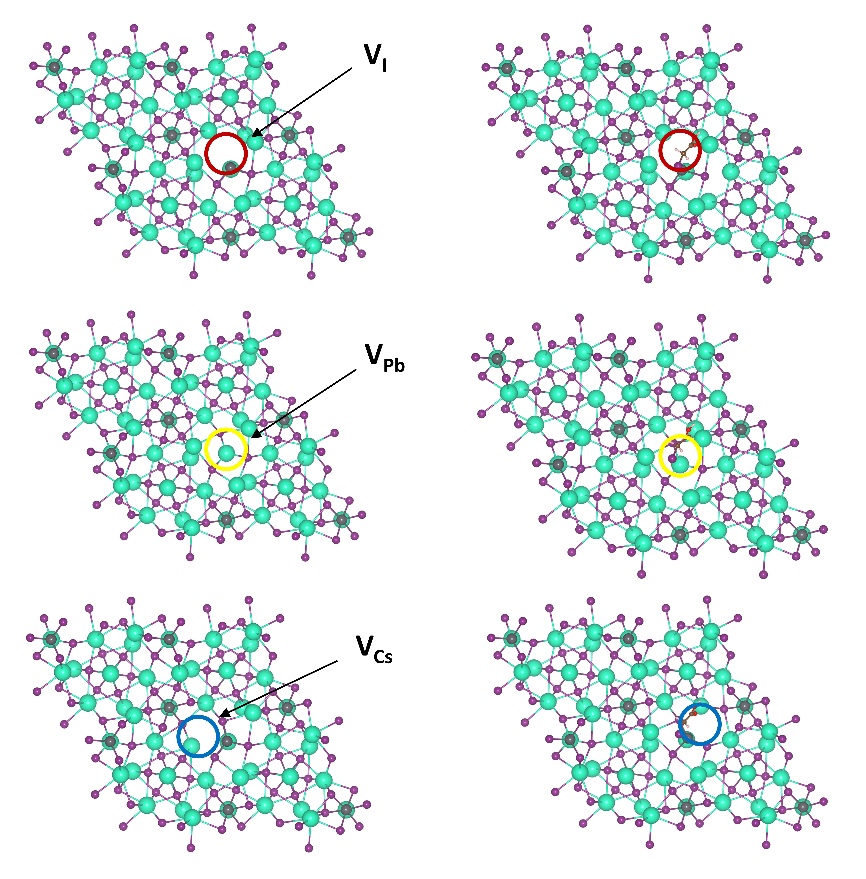
**

**Figure S4.** Cs_4_PbI_6_ crystal model on the (001) plane with V_I_, V_Pb_, and V_Cs_ defects before and after IAA regulation.

**
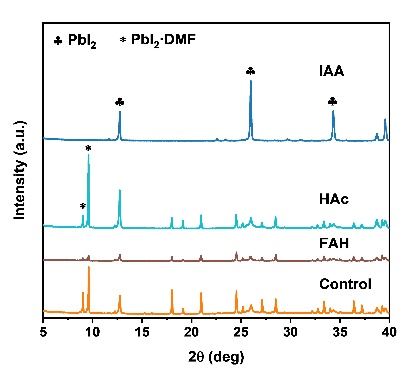
**

**Figure S5.** XRD patterns of PbI_2_-DMF powders.

**
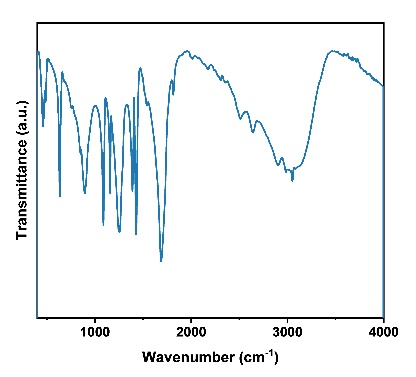
**

**Figure S6.** FTIR spectra of IAA.

**
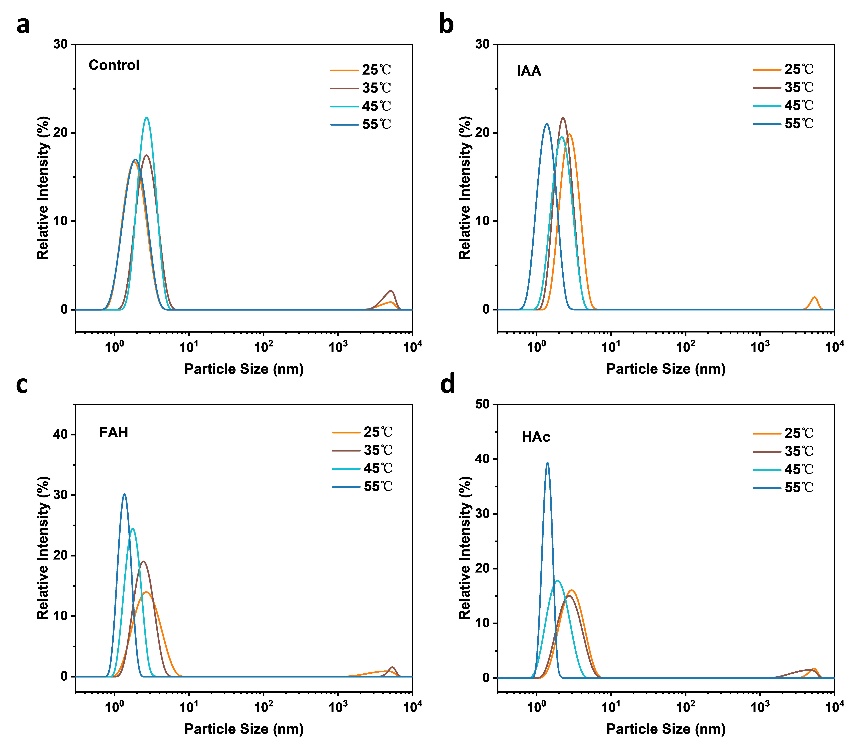
**

**Figure S7.** Temperature-dependent DLS curves of a) Control, b) FAH, c) HAc, and IAA regulated solution.

**
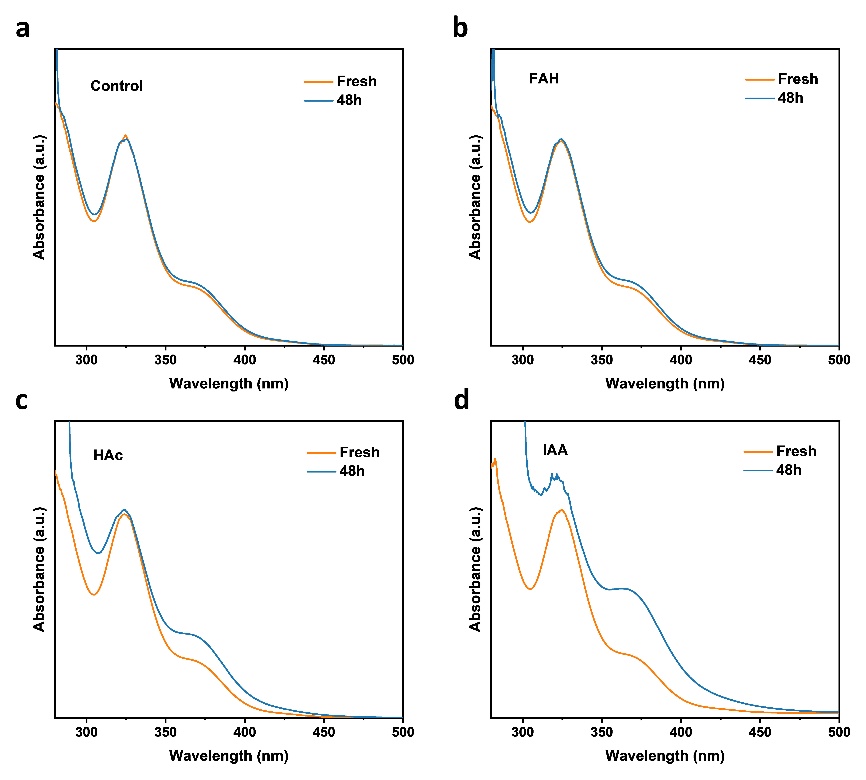
**

**Figure S8.** After aging for 48 hours, the UV-vis absorption spectra representing the evolution of characteristic peak intensity while continuously adding a) Control, b) FAH, c) HAc, and d) IAA.

**
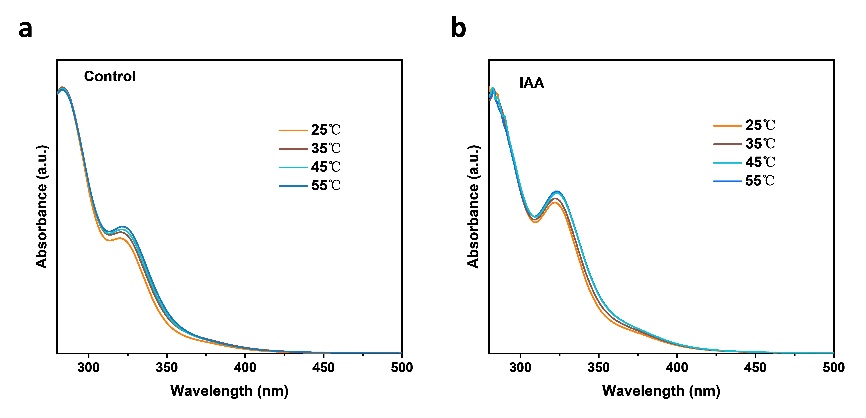
**

**Figure S9.** Temperature-dependent UV-vis spectroscopy of a) control and b) IAA regulated solution.

**
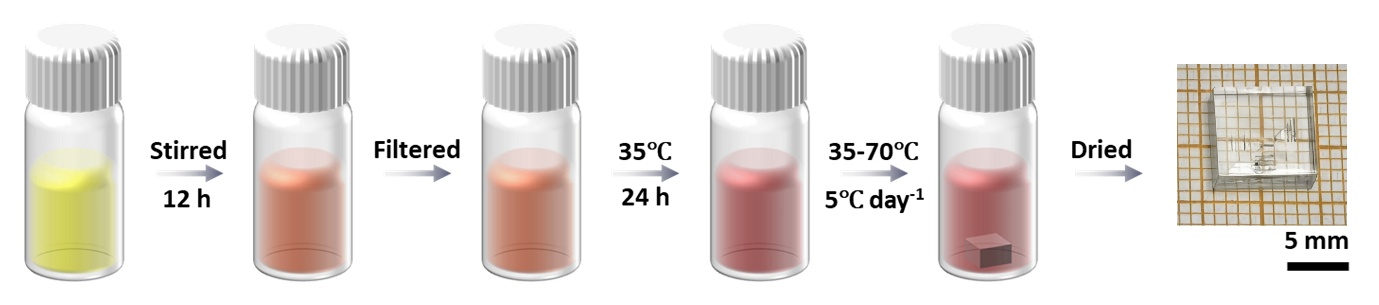
**

**Figure S10.** Schematic diagram of Cs_4_PbI_6_ single crystal grown by inverse temperature crystallization.

**
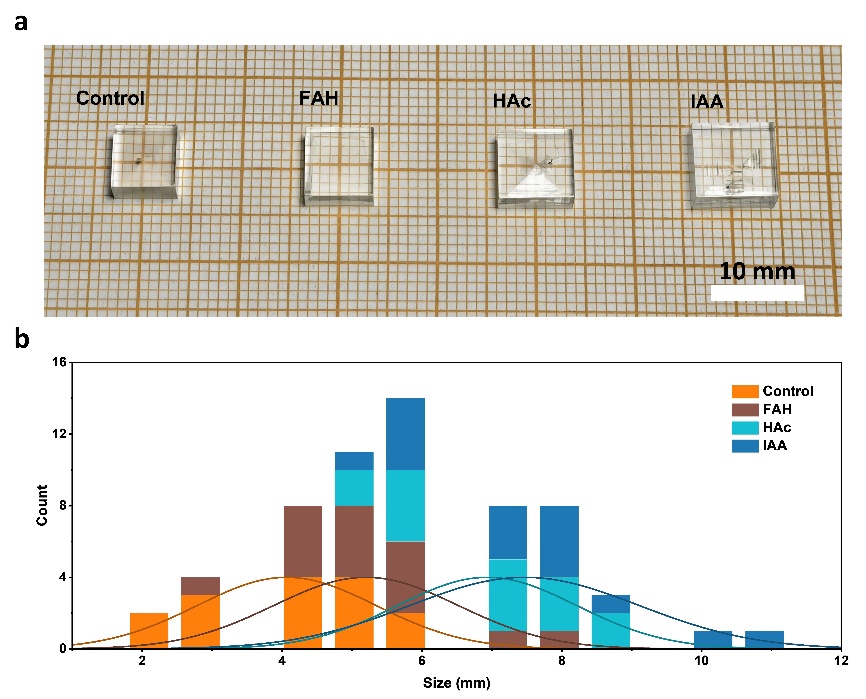
**

**Figure S11.** a) Photographs of Cs_4_PbI_6_ single crystals grown by different precursor. b) Single crystals size distribution statistical chart.

**
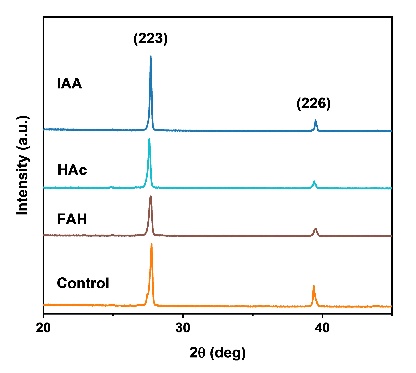
**

**Figure S12.** XRD patterns of Cs_4_PbI_6_ powders,


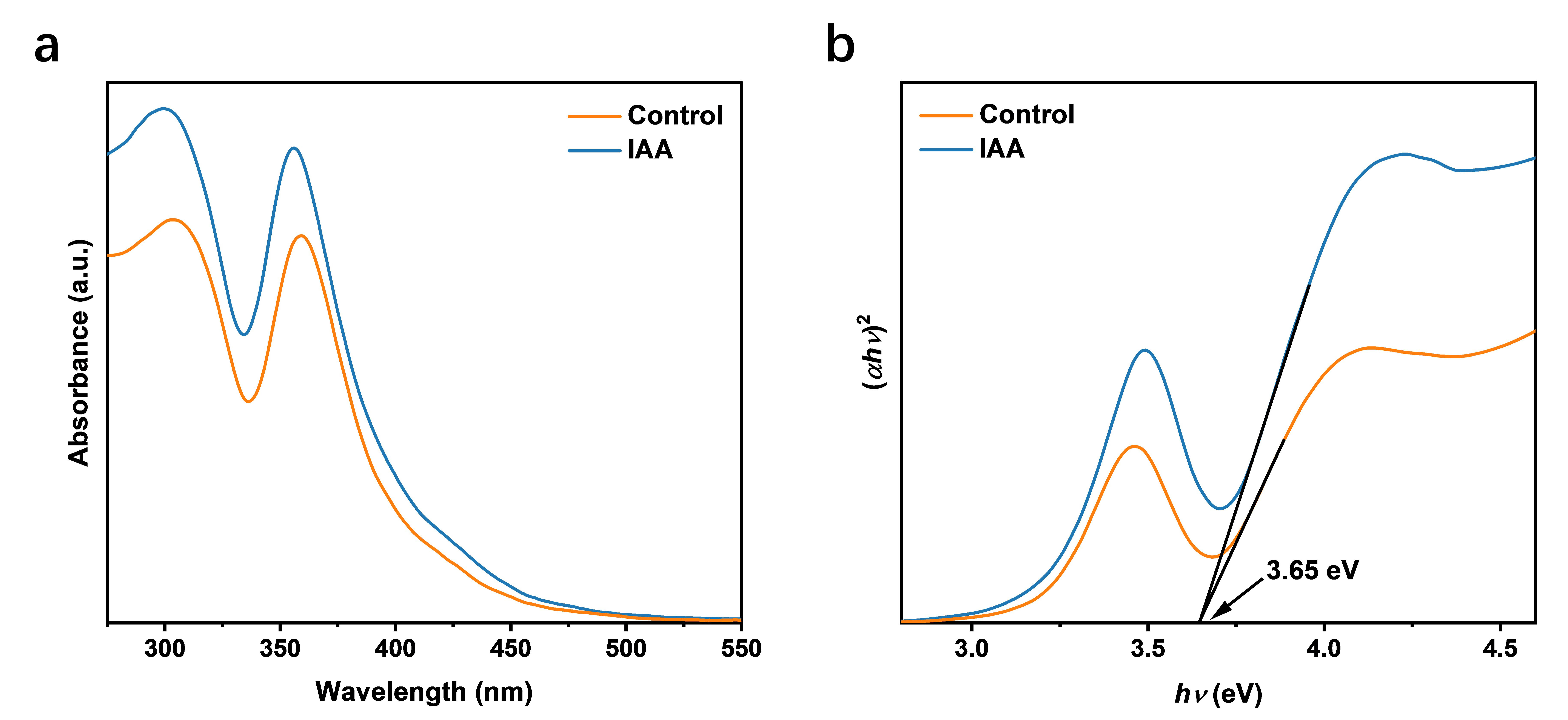


**Figure S13.** UV-vis spectroscopy and Tauc plots of control and IAA-regulated Cs_4_PbI_6_ Single crystals.

**Note.** The characteristic absorption peak appearing at 344 nm is likely caused by the Mie scattering effect resulting from the influence of powder particle size.^[6]^


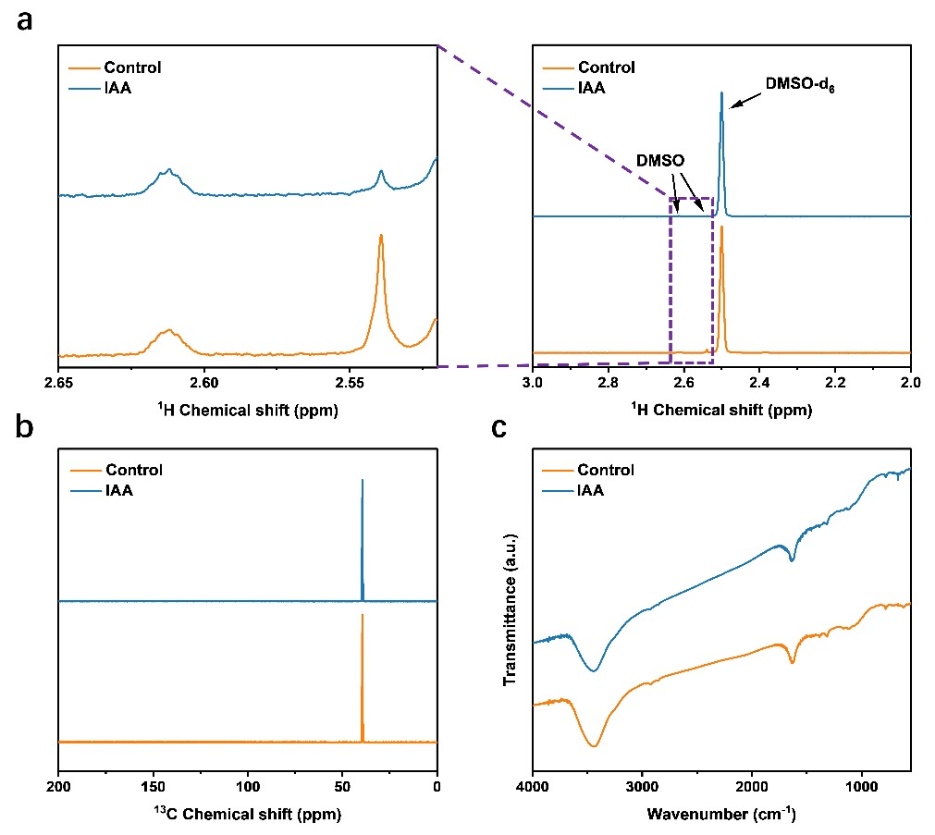


**Figure S14.** a) ^1^H NMR, b) ^13^C NMR spectroscopy and c) FTIR spectra of control and IAA-regulated Cs_4_PbI_6_ single crystals.

**Note.** In the ^1^H NMR spectrum, only the residual proton peak of DMSO-d_6_ was observed at ~2.50 ppm, along with extremely weak residual DMSO signals from the growth solvent (~2.54 and 2.61 ppm), and no discernible peaks were detected in the characteristic proton signal region of IAA. In the ^13^C NMR spectrum, only the methyl carbon signal of residual DMSO was observed at ~39.5 ppm, with no other characteristic peaks detected. By performing area integration of residual solvent peaks in the ^1^H NMR spectra of Cs_4_PbI_6_ single crystals for the control group and IAA-regulated samples, as well as elemental analysis (EA) (Table S1), it was found that the residual amounts of both solvents were significantly reduced (DMSO integration area in NMR decreased by 43.73%, N content in EA decreased by 38.61%). The FTIR spectra of the control and IAA-regulated samples were essentially consistent, with only weak absorptions observed at ~3440 cm-1 (O-H stretching, adsorbed water) and ~1633 cm-1 (H-O-H bending, adsorbed water), and no characteristic absorption peaks of IAA were observed.


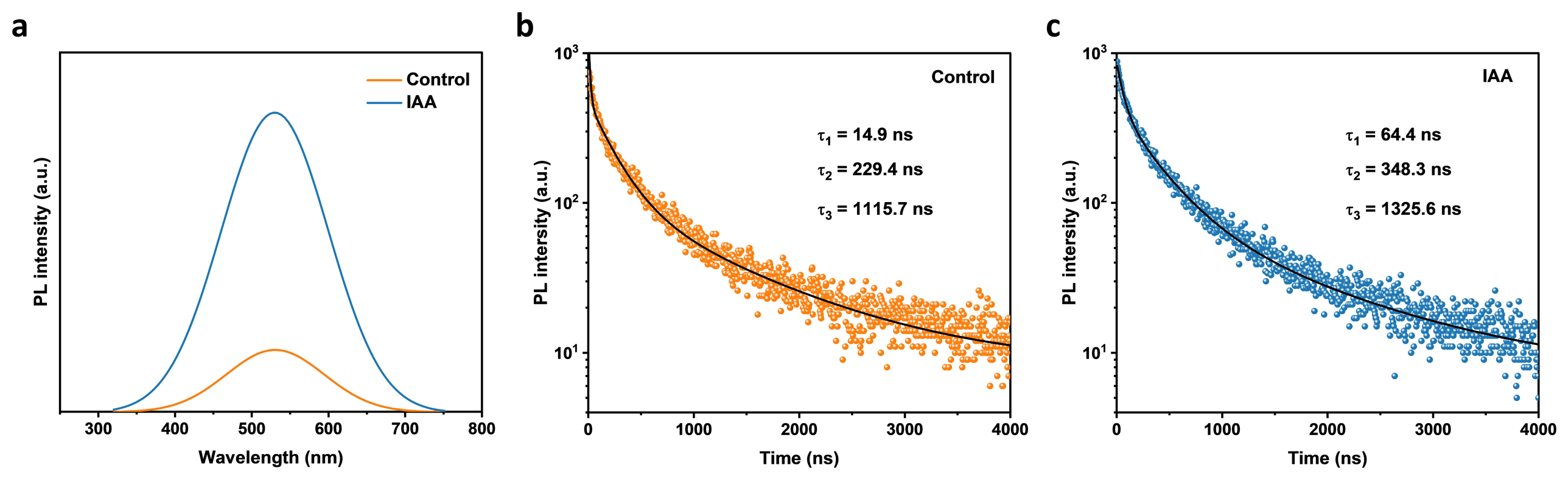


**Figure S15.** a) PL spectra of control and IAA-regulated Cs_4_PbI_6_ Single crystals. TRPL decay of b) control and c) IAA-regulated Cs_4_PbI_6_ Single crystals.

**Note.** Steady-state photoluminescence (PL) emission spectra of two sets of single-crystal materials were measured using a 265 nm excitation light source. The significant enhancement of PL intensity in IAA-regulated single crystals directly indicates that IAA treatment effectively suppresses the formation of non-radiative recombination pathways by reducing the density of defect states that act as non-radiative recombination centers. Analysis of the TRPL decay curves of Cs_4_PbI_6_ single crystals reveals that after IAA-modulated growth, the carrier recombination dynamics of the single crystals are significantly optimized. Specifically, the shortest lifetime τ_1_, representing defect-assisted non-radiative recombination, increases sharply from 14.9 ns to 64.4 ns, directly and strongly proving that IAA can effectively passivate internal crystal defects, thereby greatly suppressing non-radiative recombination pathways. Meanwhile, the intermediate lifetime τ_2_, corresponding to intrinsic radiative recombination, also increases from 229.4 ns to 348.3 ns, indicating that photogenerated carriers have a longer radiative recombination lifetime after defect elimination. The longest decay component τ_3_ rises from 1115.7 ns to 1325.6 ns, further confirming the overall enhancement of crystal quality.

**
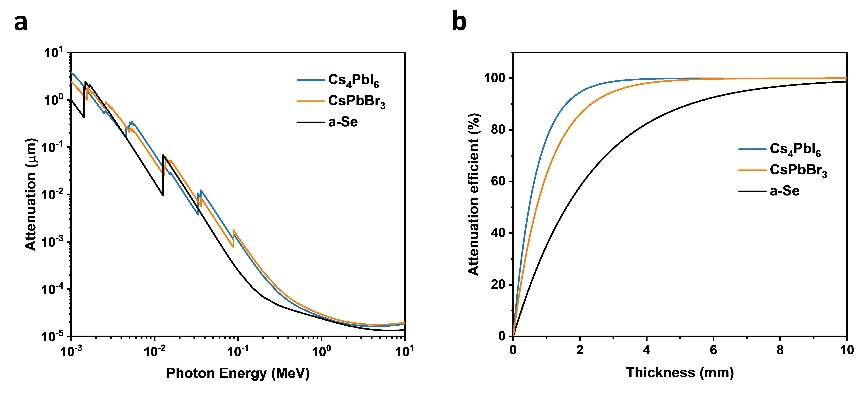
**

**Figure S16.** a) X-ray attenuation coefficient of representative semiconductors (Cs_4_PbI_6_, CsPbBr_3_, a-Se). b) Attenuation efficiency and relative thickness of Cs_4_PbI_6_, CsPbBr_3_, a-Se for 80 keV X-ray photons.

**
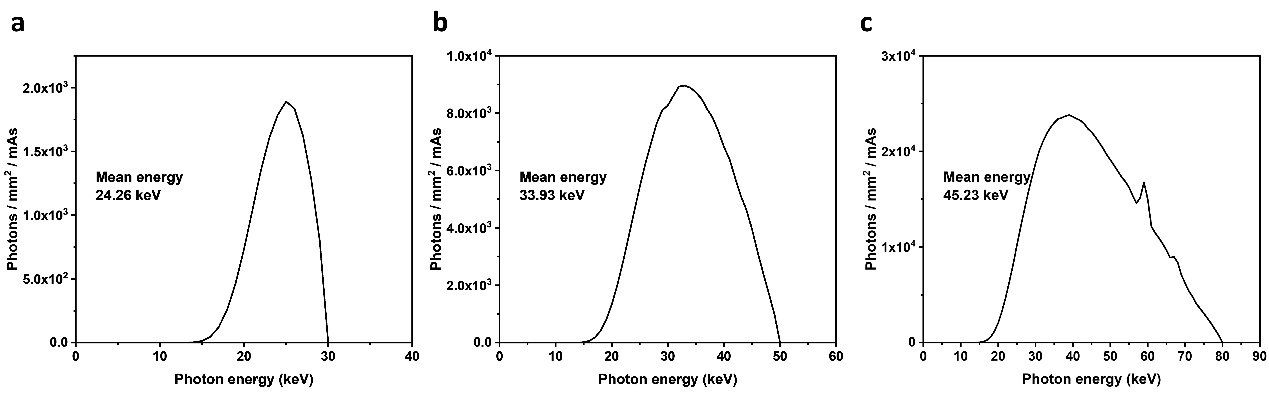
**

**Figure S17.** Mean energy of the continuum bremsstrahlung X-ray spectrum for 30, 50, 80 keV.


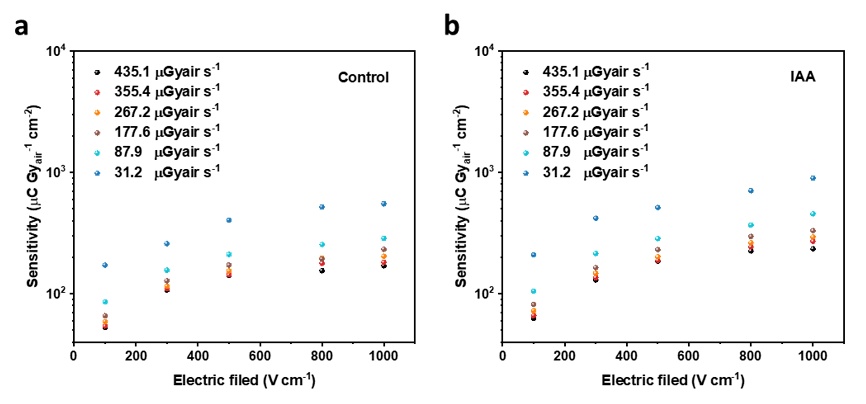


**Figure S18.** Sensitivity of the detector at different electric field potentials for a) control and b) IAA regulated Cs_4_PbI_6_.


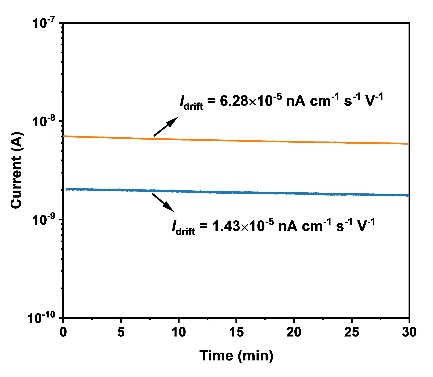


**Figure S19.** Dark current of Cs_4_PbI_6_ device under an electric field of 1000 V cm^-1^.

**Table S1**. Elemental analysis test results.

| **Sample** | **Element** | | | | | **Unit/%** |
| --- | --- | --- | --- | --- | --- | --- |
|  | **N** | **C** | **H** | **S** | **O** |  |
| Reference | 16.26 | 41.85 | 4.68 | 18.62 | 26.2 |  |
| Cs_4_PbI_6_-Control | 0.316 | 0.002 | 0.045 | 0 | 0.706 |  |
| Cs_4_PbI_6_-IAA | 0.194 | 0.017 | 0.024 | 0 | 0.513 |  |

[1] G. Kresse, J. Hafner, *Phys. Rev. B.* **1993**, 47, 558.

[2] G. Kresse, J. Hafner, *Phys. Rev. B.* **1994**, 49, 14251.

[3] J. P. Perdew, K. Burke, M. Ernzerhof, *Phys. Rev. Lett.* **1997**, 78, 1396.

[4] G. Kresse, D. Joubert, *Phys. Rev. B.* **1999**, 59, 1758.

[5] S. Grimme, J. Antony, S. Ehrlich, H. Krieg, *J. Chem. Phys.* **2010**, 132, 154104.

[6] N. Liu, R. Sun, L. Wang, Y. Ji, N. Li, B. Cao, Y. Zhang, *J. Mater. Chem. A* **2020**, 8, 5952.
